# Hepatic iron assessment using pinch liver biopsies in Asian glossy starlings (*Aplonis panayensis*)

**DOI:** 10.1177/03009858241309395

**Published:** 2024-12-30

**Authors:** Alberto Rodriguez Barbon, Charlotte Bentley, Gabby J. Drake, Miguel Mendes Veiga, Julian Chantrey, Guido Rocchigiani

**Affiliations:** 1 Chester Zoo, Chester, UK; 2 Scotland’s Rural College, Midlothian, UK; 3 University of Liverpool, Neston, UK

**Keywords:** antemortem diagnosis, hemosiderosis, iron quantification, passerine, postmortem diagnosis

## Abstract

Hepatic hemosiderosis is a common nutritional disease affecting numerous bird species. The aim of this study was to validate the use of pinch hepatic biopsies to quantify the hepatic iron in Asian glossy starlings (AGS) and compare clinical measures usable for antemortem disease monitoring. Following euthanasia, serum, liver pinch biopsies, and whole liver sections were sampled from 21 AGS. Liver biopsies and sections were stained with Perl’s Prussian blue, and the area containing iron pigment was calculated by image analysis. The mean percent iron-stained area per whole hepatic section was 62.52% ± 21.81%, while the mean percent iron-stained area in biopsies was 71.47% ± 19.77%. Positive correlations were found between hepatic iron concentration and percentage iron in whole sections (*r* = 0.92; *P* < .0001), percentage iron in biopsies (*r* = 0.91; *P* < .0001), and other clinico-pathological features. Hemosiderin in hepatic biopsies was significantly higher than in whole hepatic section assessed by image analysis (*P* < .0001).

Hemosiderosis and iron storage disease are widely described across captive and wild avian species.^3,8,12^ Hepatic biopsies, in combination with biochemical analysis of iron concentration, have been considered the gold standard for the diagnosis of iron storage disease in birds.^3,11,12^ Liver iron concentration has been assessed in avian species histologically through semiquantitative assessment of Perl’s Prussian blue (PPB)-stained tissue sections or by tissue analysis using atomic absorption spectrophotometry or inductively coupled plasma mass spectroscopy.^4,9,15,18^ In avian species, serum iron analytes have been reported to have limited diagnostic value, but serum iron and percentage of transferrin saturation have been used to assess responses to diet modification and chelation therapy.^10,20^

The Asian glossy starling (AGS; *Aplonis panayensis*) is a small frugivorous Sturnidae distributed in Southeast Asia.^5^ AGS for this study were housed in a large indoor free flight public walk-through mixed species exhibit. Birds were fed a mixed diet of chopped fruit, low iron pellets, and insects ad libitum. Routine postmortem examination of this species frequently revealed high amounts of hemosiderin in the liver.

The main objectives of this study were (1) to determine the reliability of using image analysis software for assessing hepatic iron levels via whole liver sections and pinch biopsies stained with PPB compared to tissue iron analysis and (2) to assess the correlation between clinical measures of hepatic size, serum iron, total iron binding capacity (TIBC), and percentage iron saturation with tissue iron analysis.

Twenty-one clinically healthy AGSs, including 13 females and 8 males, and based on the plumage appearance, 15 adults and 6 juveniles, were euthanized due to captive population management by administration of isoflurane overdose. Ethics was approved by the internal zoo ethic committee. A blood sample was obtained from 19 individuals by cardiocentesis. Serum was obtained after centrifugation and stored at –80°C for 12 months prior to analysis. Birds were weighed and the length of the liver silhouettes caudal to the keel were measured.^1^ A postmortem examination was carried out within 2 hours following the death, grossly examining all organs. Prior to organ dissection, a liver biopsy was obtained from the caudal margin of the right hepatic lobe using a 5 Fr flexible double action biopsy forceps (Karl Storz 67161 Z, Karl Storz Endoscopy, Slough, UK). The liver was excised, and the weights and lengths of each hepatic lobe were measured. The weight of liver as a percentage of the body weight was calculated (% H weight). A longitudinal section through the whole length of each hepatic lobe was obtained. The tissues (pinch biopsies and liver section) were placed in 10% buffered formalin. The remaining liver tissue was stored at −20°C prior to submission for iron analysis.

Following fixation for 48 hours, hepatic sections and hepatic biopsies were routinely processed for histology and paraffin embedded to produce 4-μm-thick sections. Hepatic sections and biopsies were mounted in different slides and stained with hematoxylin and eosin and PPB. Slides were scanned using a 40× objective (Aperio CS2, Leica, Milton Keynes, UK—resolution 0.25 μm/pixel).

Hematoxylin and eosin slides were examined for lesions associated with the presence of iron. PPB slides from the biopsies and whole hepatic section from both hepatic lobes were used to analyze the area occupied by iron pigment in the tissue section through image analysis software (Orbit, Manuel Stritt, Allschwil, Switzerland). A machine learning model was created and trained under the supervision of a board-certified pathologist on 50 annotations for each of the following categories: iron, identified by any blue granular material in hepatocytes and Kupffer cells; non-iron tissue, any tissue not blue pigmented; and background, optically empty spaces including empty areas of the slide and empty luminal spaces in blood vessels or bile ducts (Fig. 1c, Supplemental Figure S1). Annotations and training steps were repeated until the model was able to distinguish such categories; the model accuracy was assessed manually by reviewing the overlying mask. The area percentage with iron pigment in the whole hepatic section (%Fe WS) and the biopsy (%Fe BX) was recalculated following the exclusion of the background category. Two additional serum samples not from birds in this study were used from the zoo’s biobank, one from an AGS also stored at −80°C for 12 months before testing and one from a Cabot’s tragopan (*Tragopan caboti*) sampled and stored at −80°C for only 1 month prior to testing.

**Figure 1.**
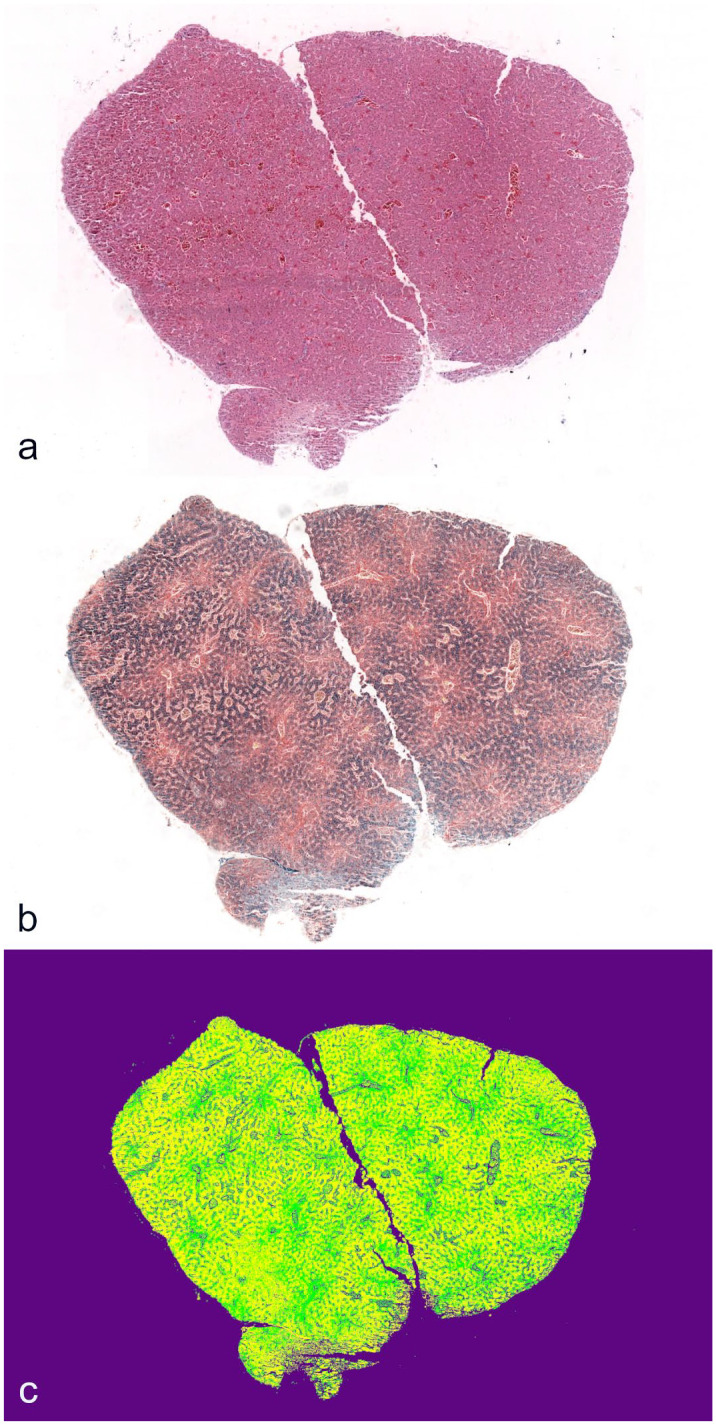
Hepatic biopsy of an Asian glossy starling. (a) Hematoxylin and eosin. (b) Perl’s Prussian blue demonstrating iron distributed throughout the liver. (c) Visualization of the same image in (b) with an overlay of the trained image analysis model. Purple, background; yellow, iron; and green, non-iron tissue.

The hepatic iron concentration ([HFe]), TIBC, and subsequent calculation of percentage saturation methodology is reported in the Supplemental Materials. Jamovi statistical software (The Jamovi Project, https://www.jamovi.org) was used for all statistical analysis. Descriptive statistics were calculated for [HFe], serum iron, body weight, liver weight, % H weight, and liver silhouette including Shapiro-Wilk normality. Mann-Whitney *U* analysis was used to assess the effect of age, group, and sex on hepatic iron measurements. Spearman’s correlation coefficients were used to analyze correlation between [HFe] and %Fe BX, %Fe WS, total serum iron, liver silhouette length, and liver weight percentage to body weight. %Fe BX was compared to %Fe WS using a Bland-Altman plot.

There were no gross or histological hepatic lesions associated with the presence of iron pigment in any of the cases.

The detailed results are presented in Supplemental Table S1. Mean [HFe] was 315,885 ± 160,775 µmol/kg dry matter (*n* = 21, range: 77,650–535,533). The mean %Fe WS was 62.52% ± 21.81% (*n* = 21, range: 19.71–89.29), and the mean %Fe BX was 71.47% ± 19.77% (*n* = 21, range: 17.92–94.49). There was a significant difference (*P* < .0001) between %Fe WS and %Fe BX. The Bland-Altman analysis showed a positive bias toward %Fe BX of 8.95% (Supplemental Figure S2, Supplemental Table S2).

The mean liver weight was 1.67 ± 0.44 g (*n* = 21, range: 2.53–1.02), and the mean liver silhouette length was 13.19 ± 2.05 mm (*n* = 21, range: 16.34–9.53). [HFe] had very strong positive correlations with %Fe WS (0.92, *P* < .01) and %Fe BX (0.91, *P* < .001), strong correlations with total serum iron (0.73, *P* < .01) and hepatic weight (*r* = 0.67; *P* < .001), and moderate positive correlations with the percentage of liver weight in relation to body weight (*r* = 0.58; *P* = .006) and liver silhouette length (0.50, *P* = .023) (Fig. 2). Adults had significantly higher %Fe WS (*P* = .018), %Fe BX (*P* = .014), and [HFe] (*P* = .018) than juveniles. Sex had no significant effects on [HFe], %Fe WS, and %Fe BX.

**Figure 2.**
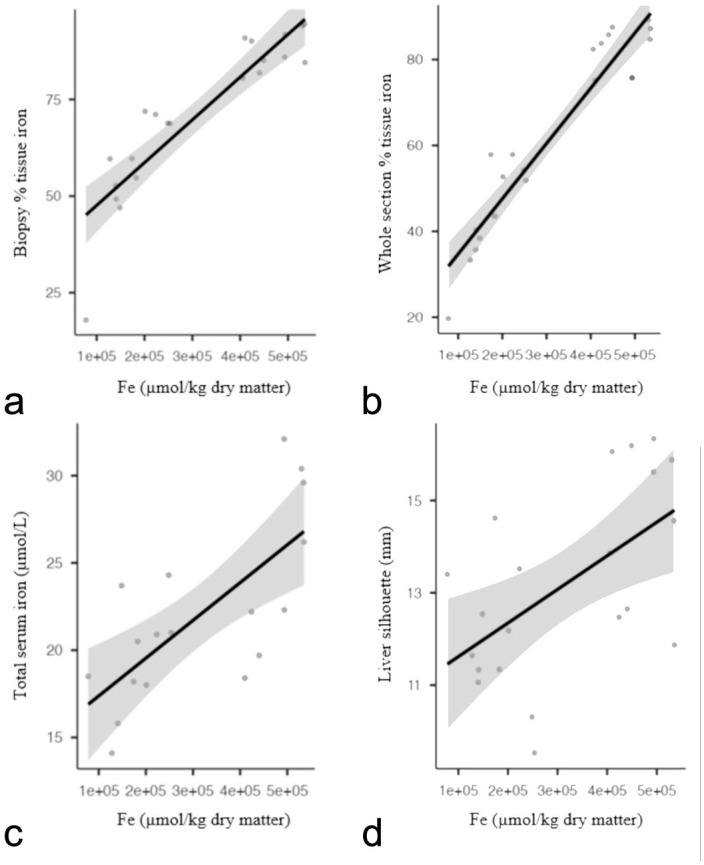
Correlation between hepatic iron concentration (Fe µmol/kg dry matter) and the percentage of iron-stained tissue in (a) hepatic pinch biopsy, (b) whole hepatic section, (c) total serum iron (µmol/L), and (d) length of the liver silhouette (mm). Hepatic iron concentration was positively associated with all other 4 features. Correlations of iron concentration were very similar among the percentage of iron-stained tissue of whole hepatic section and in pinch biopsies.

Mean total serum iron was 22.0 ± 4.99 µmol/L (*n* = 18; range: 14.1–32.1). No results were obtained in the sample where the volume was below 0.1 ml. Female birds had significantly lower total serum iron than males (*P* = .004).

From the initial batch of 18 sera, TIBC analysis yielded results below the limit of detection for all samples except in 3 cases with measurable, but low TIBC with mean 3.1 ± 1.97 µmol/L (*n* = 3; range: 1.5–5.3 µmol/L). Transferrin saturation was consequently above 100% for these 3 sera. To further clarify the source of TIBC failings, 2 additional sera from the zoo’s biobank were tested. The additional AGS stored for 12 months at −80°C also had TIBC below the detection limit; however, the Cabot’s Tragopan sera, only stored at −80°C for a month, had serum iron of 16.8 µmol/L and TIBC of 30.3 µmol/L, corresponding to transferrin saturation of 55%, a biologically meaningful result. It was therefore concluded that sample storage had adversely affected the TIBC assay.

Pinch biopsies in combination with PPB staining and image analysis of histological sections appears to be a suitable method to quantify the amount of hepatic iron in AGS. Due to the small amount of tissue collected in a pinch biopsy, this type of sample is unlikely to be suitable for analytical iron analysis, making accurate histological analysis a valuable tool for assessment of iron levels. In terminal studies in European starlings (*Sturnus vulgaris*), the minimum of liver tissue was 0.5 g when measuring non-heme iron by atomic absorption spectrophotometry.^16^ In this study, a smaller amount of tissue was used for the determination of hepatic iron concentration (0.22 ± 0.04 g, range: 0.26–0.13 g), but the amount was still considerably larger than the tissue that can be obtained with a pinch biopsy. In a previous study, 0.5 to 0.75 g of wet tissue wedge biopsies, requiring a midline coelotomy, were used to quantify iron by image analysis in pigeons (*Columba livia*),^14^ but the use of an endoscopic pinch liver biopsy can serve the same purpose, offering the possibility to obtain samples endoscopically and to sample smaller species or conduct repeat monitoring.

The use of image analysis to quantify the amount of iron in avian species has been previously described in the literature. In contrast to the use of the whole sections described in this study, these previous studies tend to use a limited number of fields in the section.^4,7,8,14,18^ In any case, regardless of the methodology, some of these studies show good correlation between the hepatic iron concentration and the image analysis results,^4,8^ as it occurs in this report.

Semiquantitative scoring (0–3) has been used in previous studies.^20^ The use of a quantitative image analysis instead of a scoring system appears more suitable and more objective to provide a sequential evaluation of biopsy specimens and to assess the response to chelation therapy of dietary modification, as shown previously in humans and avian species.^2,13^

The iron percentage area in whole sections (62.52% ± 21.81%) and biopsies (71.47% ± 19.77%) showed marked variability. In some clinically healthy AGS that were free of hepatic lesions in this study, the percentage affected area was higher than the reported affected area in other studies. For instance, an individual bared face curassow (*Crax fasciolata*) with a 42.9% affected area, and a channel-billed toucan (*Ramphastos vitellinus*) with 66.55%, showed clinical disease and hepatic histological lesions associated with the iron accumulation,^8^ where the AGS did not. Previous research evaluating areas of hepatic iron deposition across multiple species suggest that the area affected by iron deposits is not predictive of the presence of clinical disease,^3,4,7^ and it is likely that significant variation in the hepatic iron tissue content occurs across different species. This finding emphasizes the need for histological evaluation for hepatic lesions in association with iron accumulation when the clinical presentation is suggestive of hepatic disease.

Despite the strong correlation in both biopsy and whole section with the iron concentration, there was a significant difference between the 2 samples in the iron quantification. This may be the result of some degree of tissue compression while obtaining the pinch biopsy or uneven distribution of iron through the liver, with the periphery of the organ potentially being higher, where the pinch biopsy was obtained.

[HFe] in AGS observed in this study (315,885 ± 160,775 µmol/kg dry matter) was markedly higher than the results observed in other studies evaluating Sturnidae fed experimentally high iron diet. European starlings fed a high iron diet (28,380 µmol/kg dry matter) for 16 weeks showed [HFe] ranging from 25,479 to 33,075 µmol/kg dry matter,^16^ while in a different study using the same species fed a diet with 54,342 µmol/kg dry matter, the [HFe] was 106,159 ± 16,777 µmol/kg dry matter.^6^ Regardless of the absence of clinical disease or hepatic lesion in AGS with the reported [HFe], caution should be exercised in using the results of this study as a reference for other species as seasonality and female reproductive status are both factors shown previously to affect levels of iron in other avian species,^17,19^ which have not been evaluated here.

In this study, serum iron showed a strong correlation to [HFe] suggesting its potential use as a noninvasive clinical monitor of hepatic iron accumulation in AGS. We also found that samples stored for prolonged periods, even at −80°C, cannot be relied upon for quantitative colorimetric TIBC analysis, and future work should ensure samples are analyzed as soon as possible after collection.

In summary, the findings support the use of hepatic pinch biopsies and serum iron as useful tools for quantitative evaluation of the amount of hepatic iron in a small passerine species, allowing accurate measurement of response to chelation therapy.

## Supplemental Material

sj-pdf-1-vet-10.1177_03009858241309395 – Supplemental material for Hepatic iron assessment using pinch liver biopsies in Asian glossy starlings (Aplonis panayensis) Supplemental material, sj-pdf-1-vet-10.1177_03009858241309395 for Hepatic iron assessment using pinch liver biopsies in Asian glossy starlings (Aplonis panayensis) by Alberto Rodriguez Barbon, Charlotte Bentley, Gabby J. Drake, Miguel Mendes Veiga, Julian Chantrey and Guido Rocchigiani in Veterinary Pathology
