# Supplementary material for: Hepatic iron assessment using pinch liver biopsies in Asian glossy starlings (Aplonis panayensis)
Source: Vet Pathol. 2024 Dec 30;62(3):360–3. doi: 10.1177/03009858241309395 (PMC12014956; doi:10.1177/03009858241309395)
Supplement: sj-pdf-1-vet-10.1177_03009858241309395 – Supplemental material for Hepatic iron assessment using pinch liver biopsies in Asian glossy starlings (Aplonis panayensis) [file sj-pdf-1-vet-10.1177_03009858241309395.pdf]

Supplemental Materials

Hepatic iron assessment using pinch liver biopsies in Asian glossy starlings (*Aplonis panayensis*)

Alberto Rodriguez Barbon, Charlotte Bentley, Gabby J. Drake, Miguel Mendes Veiga, Julian Chantrey, Guido Rocchigiani.

Supplemental Materials and Methods

The hepatic iron concentration ([HFe]) was analyzed by the following methodology. Samples were freeze dried to constant weight (Modulyo, Edwards Crawley, UK) to approximately 0.1-0.2 g (0.22 ± 0.04 g, maximum 0.26 g, minimum 0.13 g) of dry material accurately weighed into a high pressure digestion vessel (HVT50, Anton Paar, St Albans, UK). 3 ml 68% nitric acid (Primar plus, Fisher Scientific, Loughborough, UK), 2 ml 30% hydrogen peroxide (Analar, VWR Ltd, Lutterworth UK), and 3ml deionized water (Chorus1, Veolia Water Technologies High Wycombe, UK. 18.2 MΩ) were added before being run on a digest microwave (Multiwavepro, Anton Parr, St Albans, UK) with a 10-minute ramp to 140°C, 20-minute hold at 140°C, and subsequent cooling to 55°C. Digested contents were transferred to a 25 ml universal tube (Sarstedt, Leicester UK) with 7 ml deionized water (as above). Blanks and appropriate standards/certified reference material were included with each batch run.

After a 1:20 dilution (0.5 ml in 10 ml) with 0.5% HNO_3 into 14 ml (105 mm x 16.8mm) polypropylene tubes (Sarstedt, Leicester UK), multi-element analysis was undertaken. This was via inductively-coupled plasma mass spectroscopy (Thermo-Fisher iCAP-Q, Thermo-Fisher Scientific, Loughborough, UK) with a ‘Flatopole collision cell’ (charged with helium gas for all elements except selenium where it is charged with hydrogen – changes within sample) upstream of the analytical quadrupole to reduce polyatomic interferences. Internal standards were introduced to the sample stream via a T-piece and typically included Sc (50 µg L-1), Ge (20 µg L-1) Rh (10 µg L-1), and Ir (5 µg L-1) in the preferred matrix of 2% HNO_3. External calibration standards were usually all in the range 0 – 100 µg L-1 (ppb) for trace elements. Samples were introduced via a covered autosampler (Cetac ASX-520) through a 1317090 pfa-st nebulizer (ESI) (Thermo-Fisher Scientific, Loughborough, UK). Sample processing was undertaken using ‘Qtegra software’ (Thermo-Fisher Scientific, Loughborough, UK).

Nineteen serum samples were initially submitted for analysis of serum iron concentration, total iron binding capacity (TIBC) and subsequent calculation of percentage saturation, using two quantitative colorimetric assays. The sample volume was between 0.1 and 0.4 ml, except in one sample with volume < 0.1 ml which could not be tested. Sera were allowed to thaw slowly at 4°C for analysis and all samples were run in the same batch together with other diagnostic samples unrelated to this study. Both assays were carried out on automated analyzer RX imola (Randox Laboratories, Crumlin, County Antrim, United Kingdom) using the respective RX series reagents and following manufacturer’s instructions. In summary, iron was dissociated from transferrin and converted to its ferrous form and subsequently complexed with chromogen. This resulted in a colored chromophore with a color intensity directly proportional to serum iron concentration. For total iron binding capacity, the dissociation of iron from transferrin was done in medium

containing chromazurol B (an iron-binding dye) and ferric chloride allowing the formation of a colored dye-iron complex. A neutral buffer was subsequently added, and the resulting rise in pH caused an increased affinity of transferrin to iron, dissociating it from the dye-iron complex. Absorbance of the dye-iron complex was measured in both steps and the respective decrease was directly proportional to TIBC. For further comparison, two additional sera were tested. A different Asian glossy starling, which was collected February 2023, was tested for TIBC only, and a sample from a Cabot's tragopan (*Tragopan caboti*) that collected January 2024, within a month of testing, was tested for both serum iron and TIBC.

Supplemental Figures

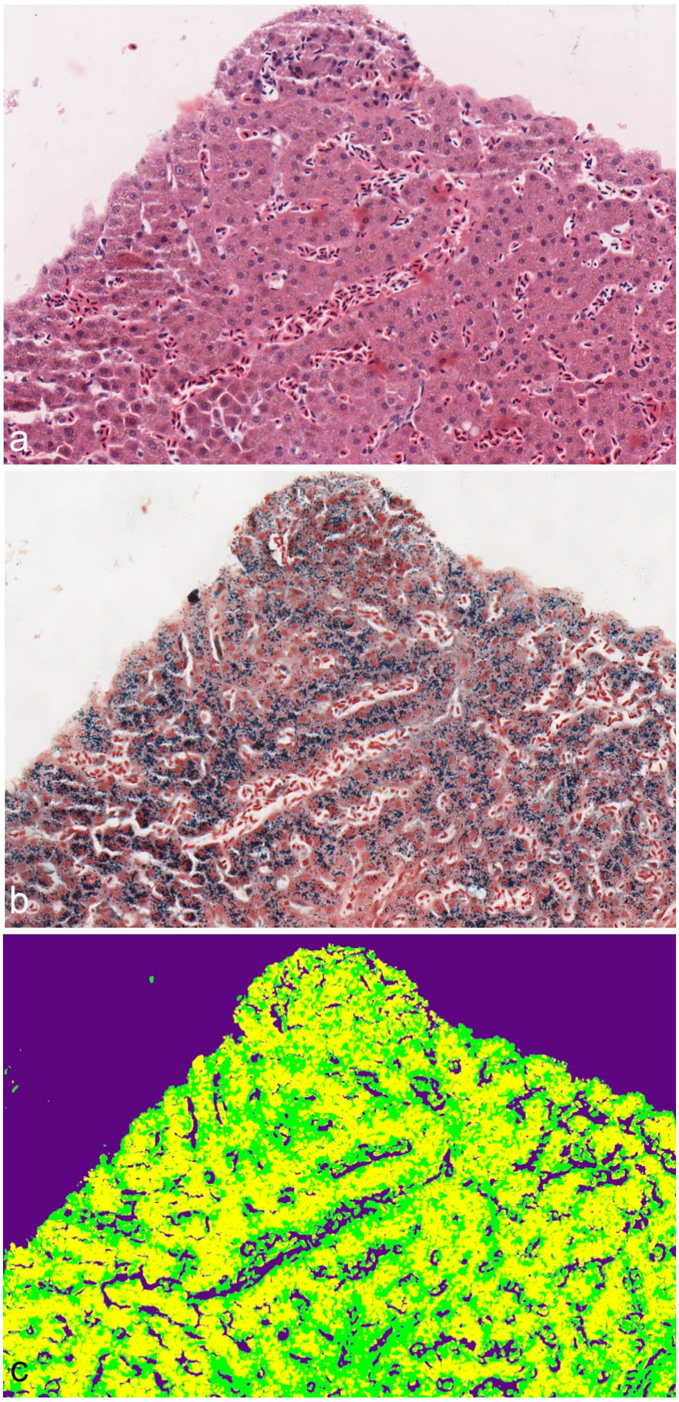

Supplemental Figure S1. Hepatic biopsy of an Asian glossy starling, higher magnification. **a)** Hematoxylin and eosin. **b)** Perl's Prussian blue demonstrating iron distributed throughout the liver. **c)** Visualization of the same image in (b) with an overlay of the trained image analysis model. Purple, background; yellow, iron; and green, non-iron tissue.

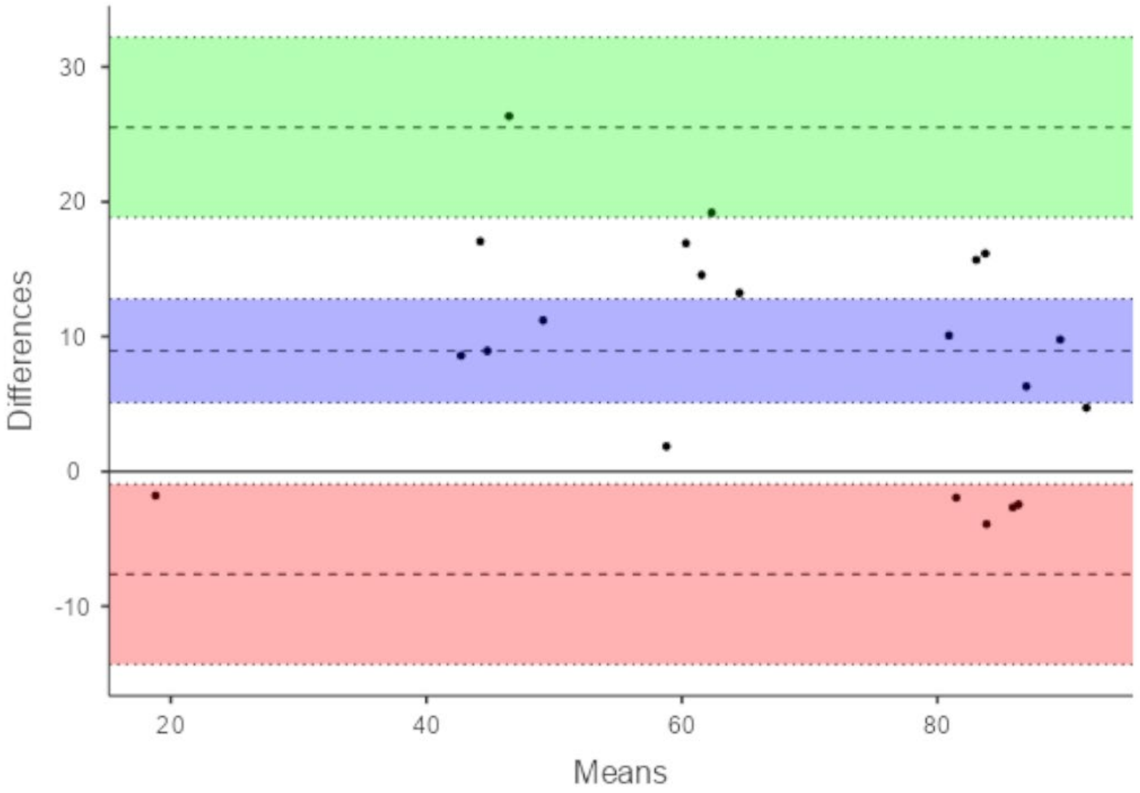

Supplemental Figure S2. Bland-Altman plot between the percentage area of iron pigment in the biopsy and percentage area of iron pigment in the whole section.

Supplemental Table S1. Descriptive statistics of each bird data

Case	Sex	Age	Animal weight (g)	Liver weight (g)	Liver % body weigth	Liver silhoutte (mm)	Serum iron (µmol/L)	[HFe] (µmol/kg DM)	%Fe WS (%)	%Fe BX (%)
1	F	A	46.9	1.02	2.2	13.4	18.5	77650	19.71	17.92
2	F	A	41.06	1.75	4.3	12.47	22.2	423946	83.79	90.10
3	M	A	43.19	1.15	2.7	12.54	23.7	148494	38.42	47.01
4	M	J	43.6	1.5	3.4	13.52	20.9	223174	57.89	71.12
5	F	A	43.29	1.16	2.7	11.34	20.5	182652	43.53	54.74
6	F	J	41.6	1.88	4.5	11.06		139771	35.69	52.75
7	F	A	42.1	1.21	2.9	14.62	18.2	173810	57.86	59.72
8	F	A	41	1.8	4.4	13.86		405503	82.43	80.49
9	F	A	46.2	2.53	5.5	16.19		449081	87.56	85.11
10	M	A	48.2	1.59	3.3	11.87	26.2	535533	87.22	84.56
11	M	A	43.8	2.2	5.0	15.88	30.4	530268	89.29	94.01
12	F	J	42.2	1.49	3.5	11.33	15.8	140779	40.30	49.24
13	M	A	46.8	1.94	4.1	16.06	18.4	410023	75.19	90.88
14	M	A	42	1.81	4.3	14.56	29.6	534625	84.71	94.49
15	F	A	44.7	2.45	5.5	16.34	22.3	493742	75.66	91.82
16	M	A	44.6	2.07	4.6	15.62	32.1	493281	75.85	85.93
17	F	A	37.9	1.15	3.0	9.53	21	253543	51.85	68.77
18	M	J	42.9	1.49	3.5	10.31	24.3	248467	54.25	68.82
19	F	A	42.4	2.02	4.8	12.65	19.7	440426	85.78	81.88
20	F	J	36.5	1.2	3.3	12.18	18	201041	52.73	71.92
21	F	J	41	1.56	3.8	11.64	14.1	127770	33.30	59.64
Mean			42.95	1.67	3.87	13.19	21.99	315884.71	62.52	71.47
Median			42.90	1.59	3.80	12.65	20.95	253543.00	57.89	71.92
SD			2.75	0.43	0.91	2.00	4.84	156900.15	21.28	19.29
Max			48.20	2.53	5.48	16.34	32.10	535533.00	89.29	94.49
Min			36.50	1.02	2.17	9.53	14.10	77650.00	19.71	17.92

Abbreviations: F, female; M, male; A, adult; J, juvenile; [HFe], hepatic iron concentration; %Fe WS, percentage area of iron pigment in the whole section; %Fe BX (%), percentage area of iron pigment in the biopsy; SD, standard deviation; Max, maximum value; Min, minimum value.

Supplemental Table S2. Bland-Altman analysis between the percentage area of iron pigment in the biopsy and percentage area of iron pigment in the whole section.

		95% confidence interval	
	Estimate	Lower	Upper
Bias (n=21)	8.95	5.1	12.797
Lower limit of agreement	-7.63	-14.31	-0.94
Upper limit of agreement	25.52	18.84	32.207
